# Supplementary figures and images for: SLC1A5 provides glutamine and asparagine necessary for bone development in mice
Source: eLife. 2021 Oct 14;10:e71595. doi: 10.7554/eLife.71595 (PMC8553342; doi:10.7554/eLife.71595)

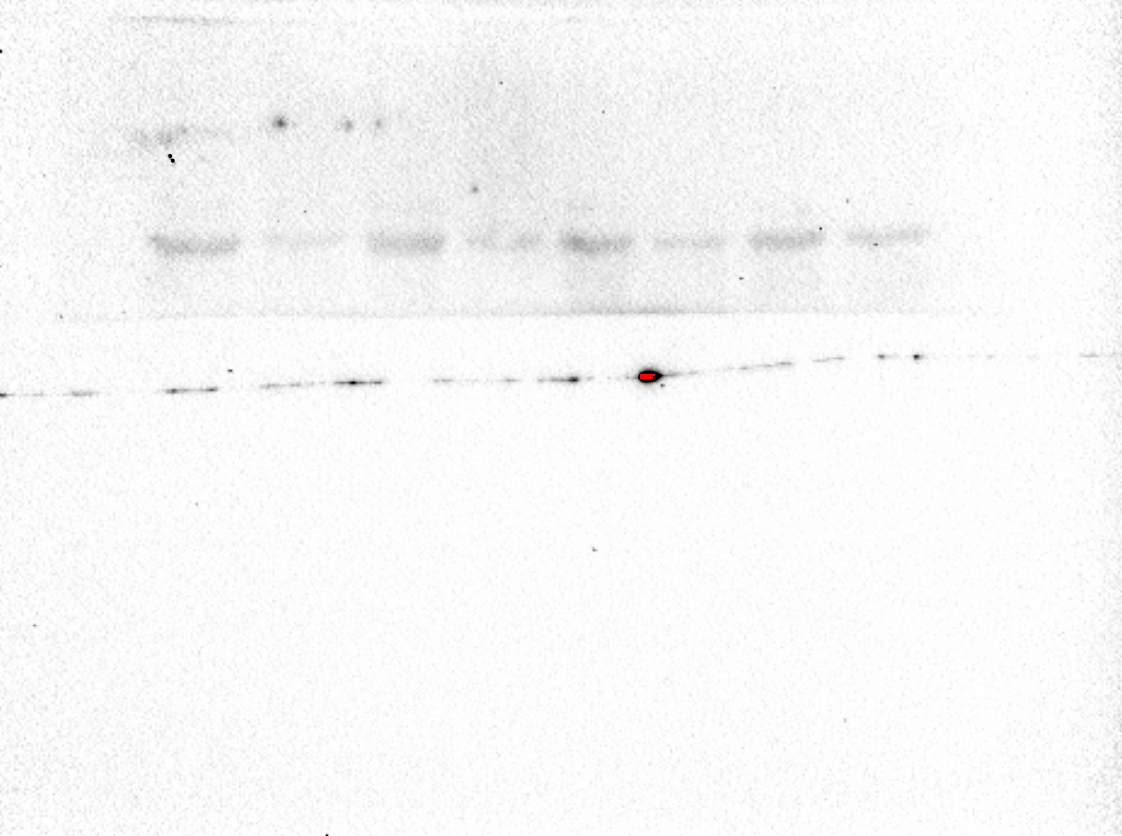

Supplement: Figure 1—figure supplement 1—source data 1. [file elife-71595-fig1-figsupp1-data1.zip › Figure 1 figure supplement 1 Source Data/Sharma Figure 1 figure supplement 1 Western Blot Source Data/Sharma Figure S1B Source file ASCT2.tif]

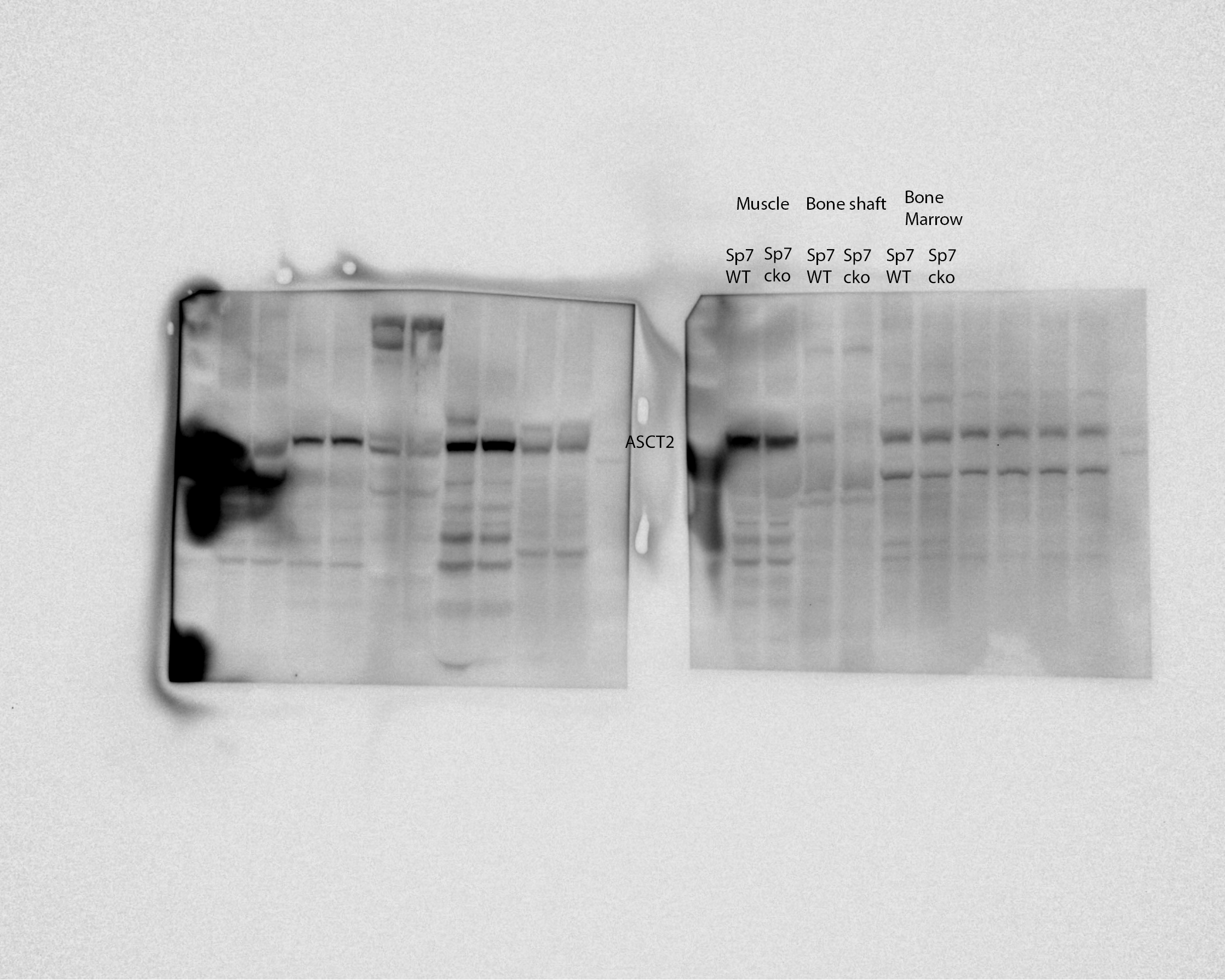

Supplement: Figure 1—figure supplement 1—source data 1. [file elife-71595-fig1-figsupp1-data1.zip › Figure 1 figure supplement 1 Source Data/Sharma Figure 1 figure supplement 1 Western Blot Source Data/Sharma Figure S1D Souce Data ASCT2.tif]

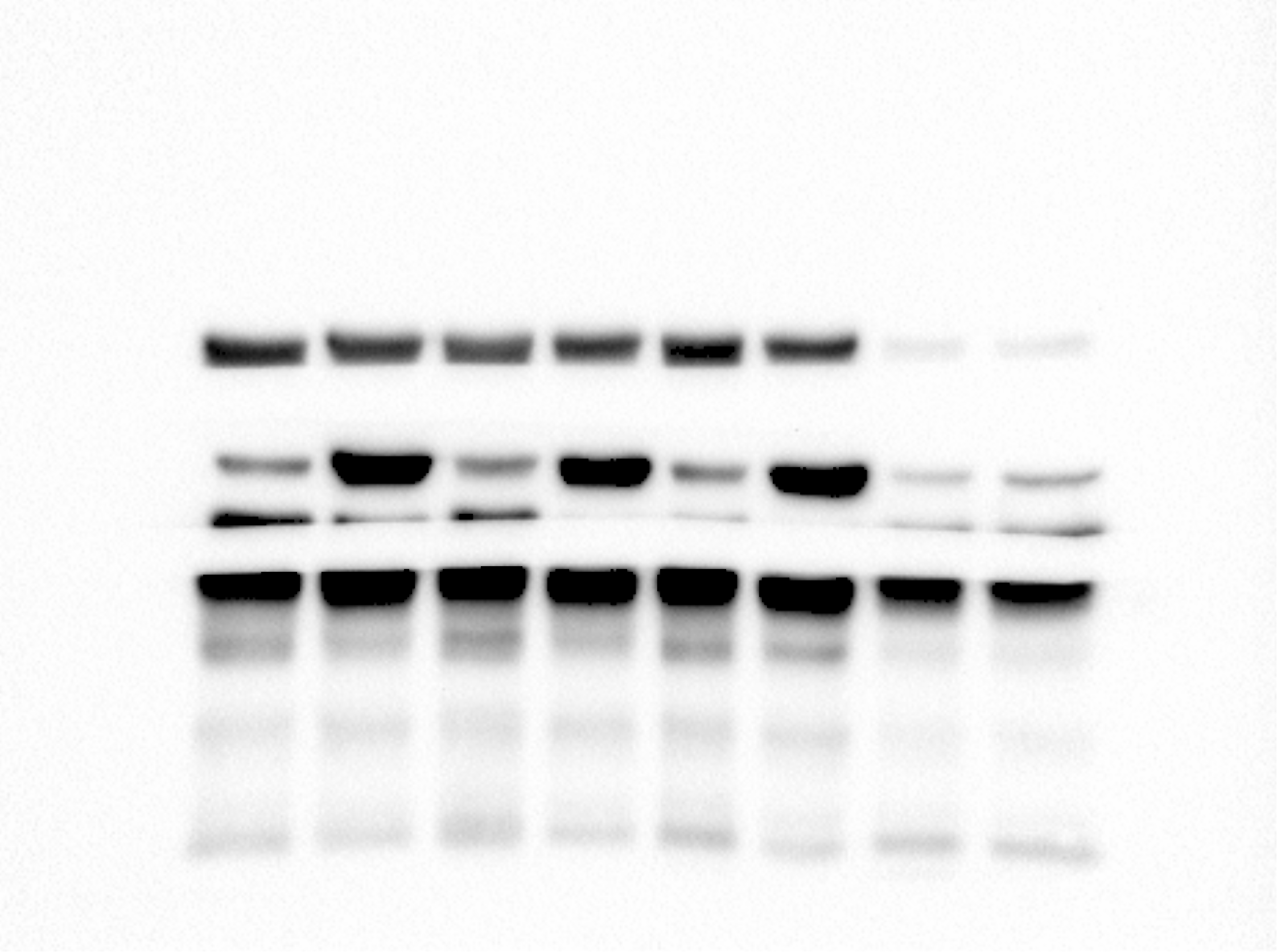

Supplement: Figure 1—figure supplement 1—source data 1. [file elife-71595-fig1-figsupp1-data1.zip › Figure 1 figure supplement 1 Source Data/Sharma Figure 1 figure supplement 1 Western Blot Source Data/Sharma Figure S1B Source file TUBULIN.tif]

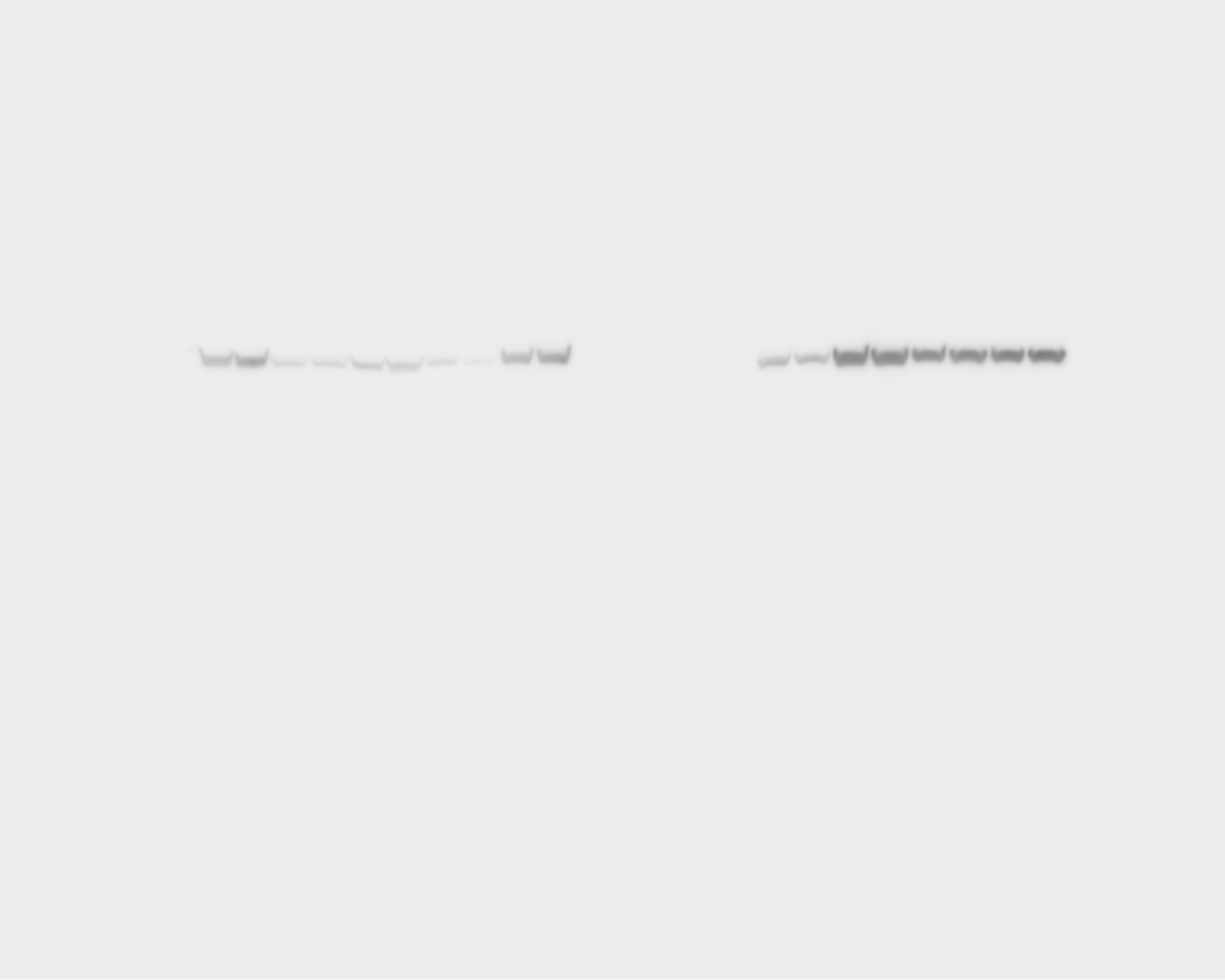

Supplement: Figure 1—figure supplement 1—source data 1. [file elife-71595-fig1-figsupp1-data1.zip › Figure 1 figure supplement 1 Source Data/Sharma Figure 1 figure supplement 1 Western Blot Source Data/Sharma Figure S1D Source file actin.tif]

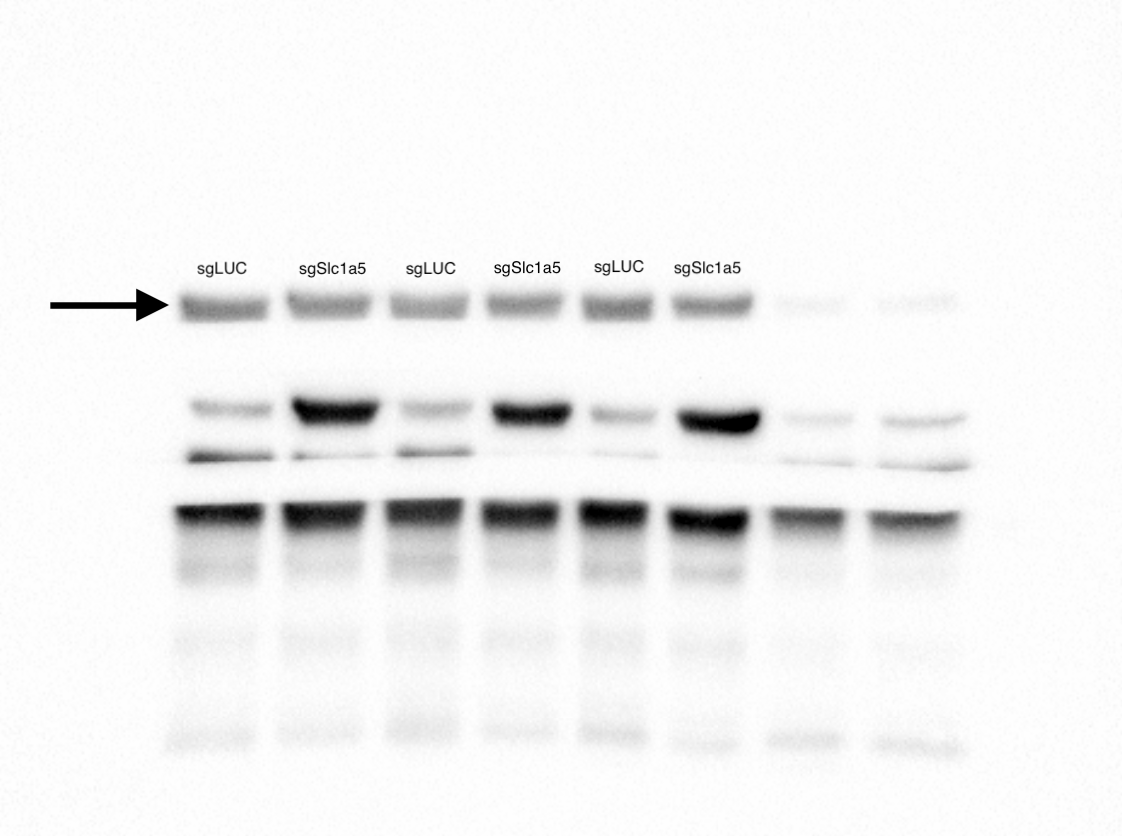

Supplement: Figure 4—source data 1. [file elife-71595-fig4-data1.zip › Figure 4 Source Data/Sharma Figure 4C Source Data/Sharma Figure 4C Source File TUBULIN.tiff]

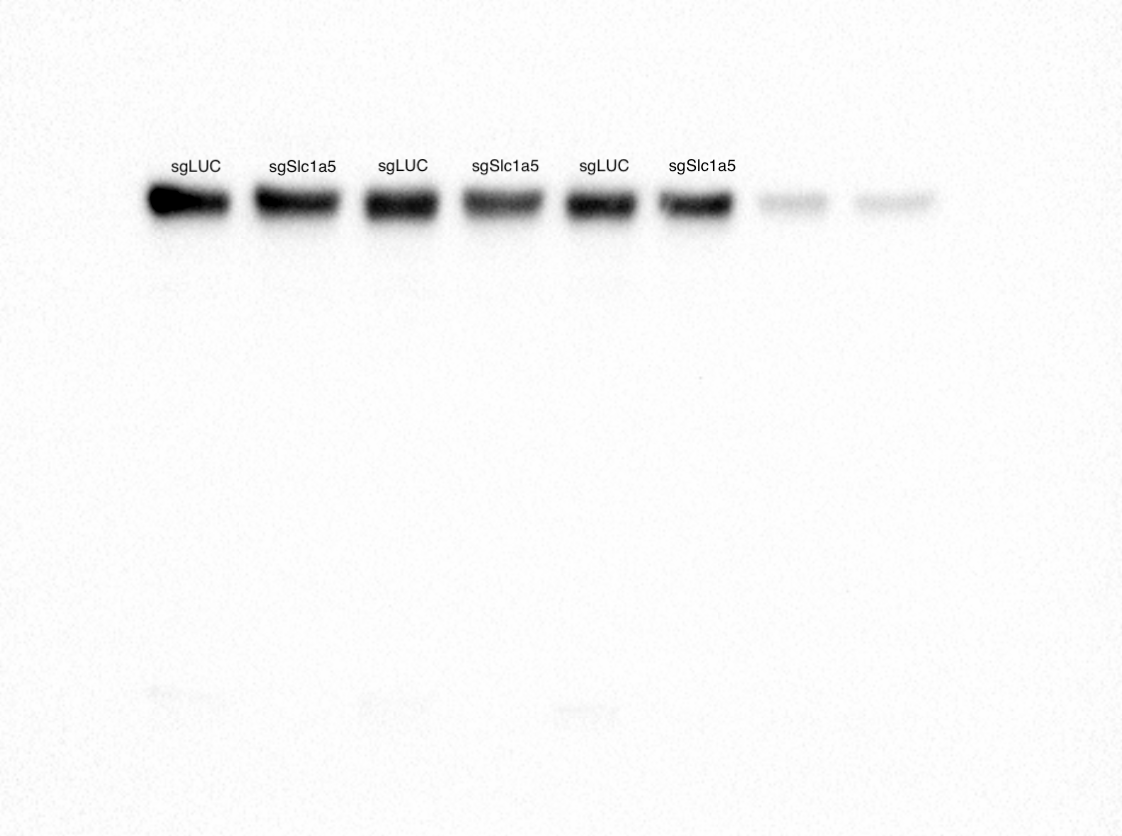

Supplement: Figure 4—source data 1. [file elife-71595-fig4-data1.zip › Figure 4 Source Data/Sharma Figure 4C Source Data/Sharma Figure 4C Source File Total S6.tiff]

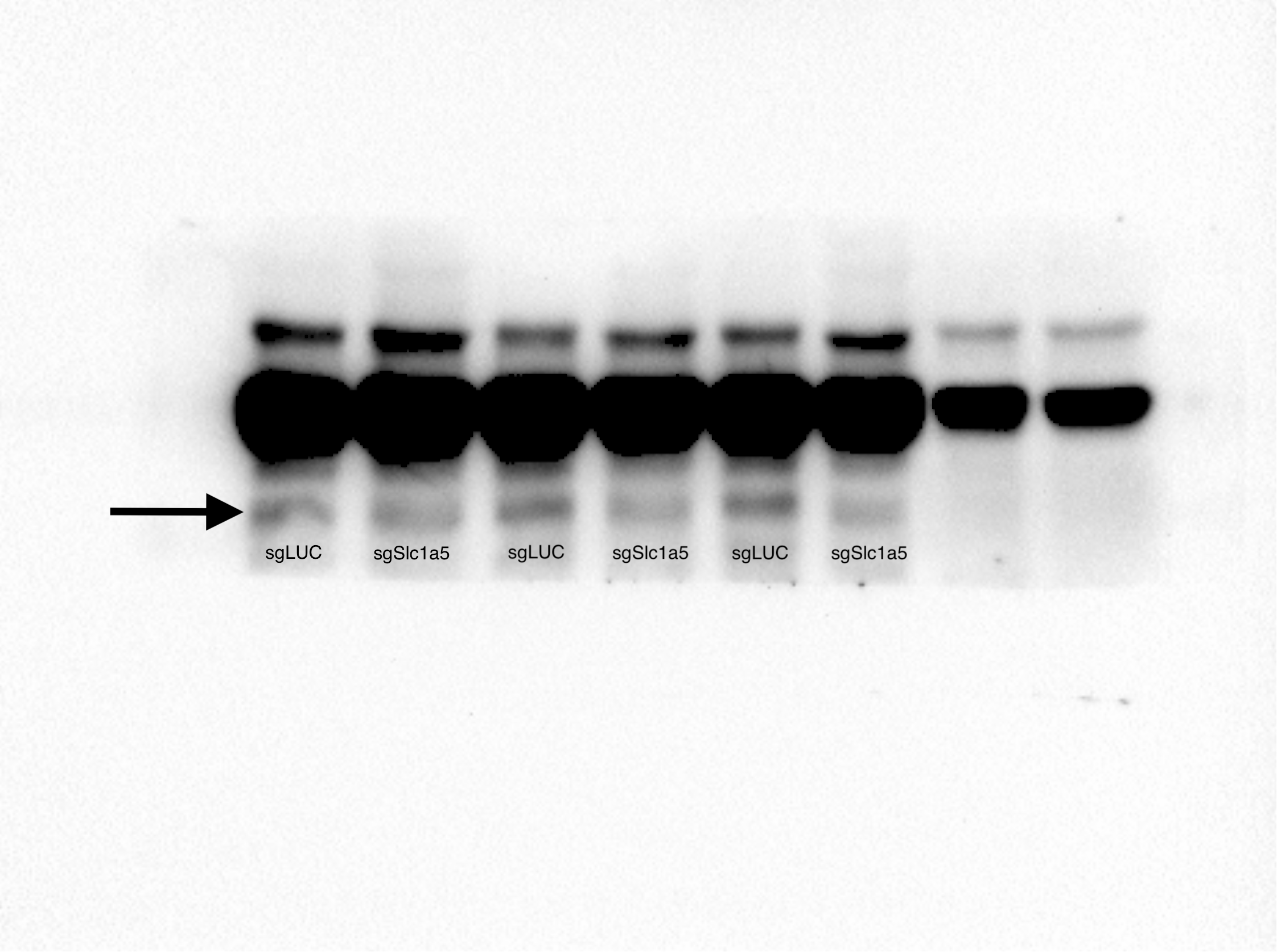

Supplement: Figure 4—source data 1. [file elife-71595-fig4-data1.zip › Figure 4 Source Data/Sharma Figure 4C Source Data/ Sharma Figure 4C Source File phospho 4EBP1.tiff]

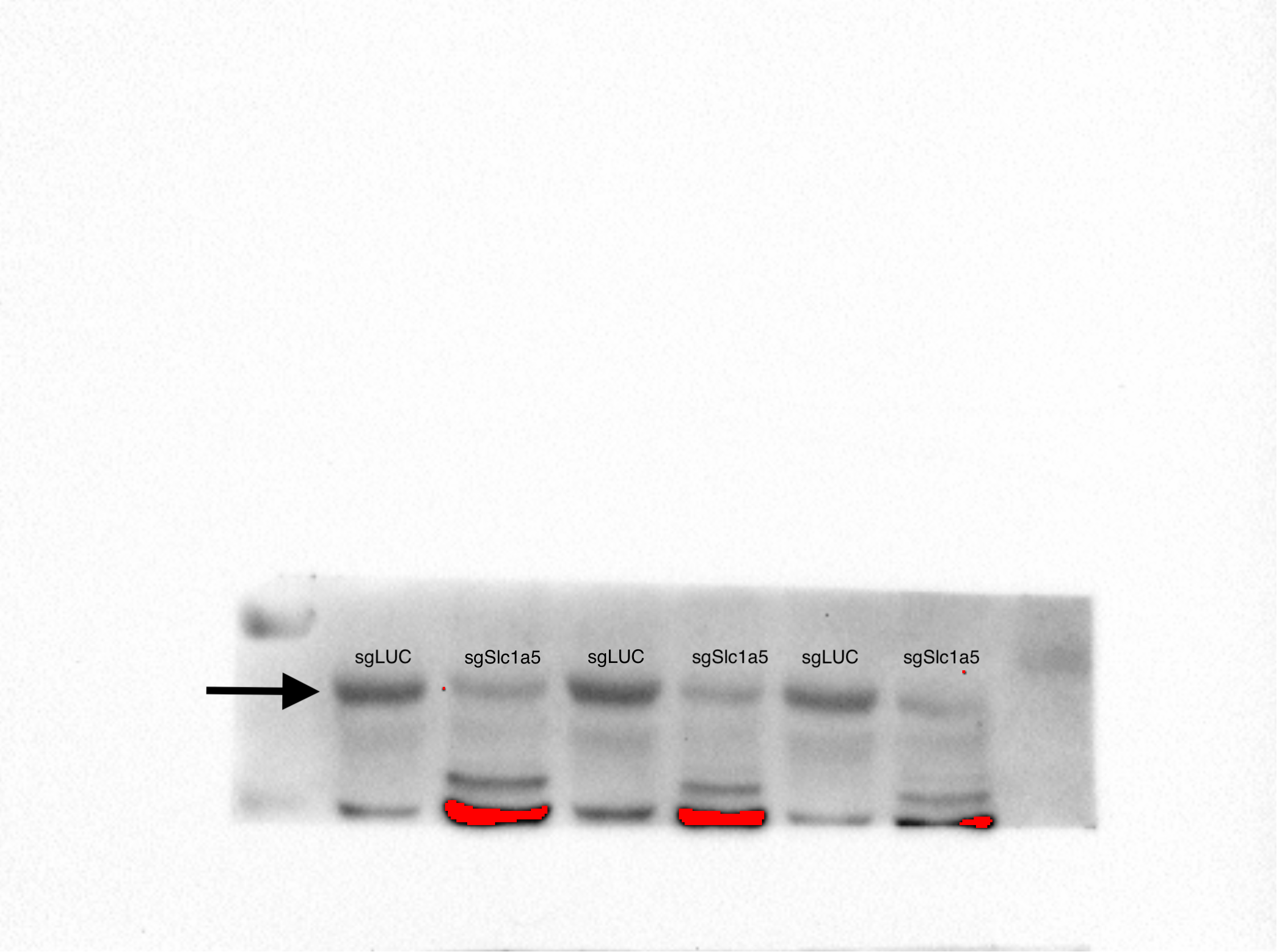

Supplement: Figure 4—source data 1. [file elife-71595-fig4-data1.zip › Figure 4 Source Data/Sharma Figure 4C Source Data/Sharma Figure 4C Source File ASCT2 .tiff]

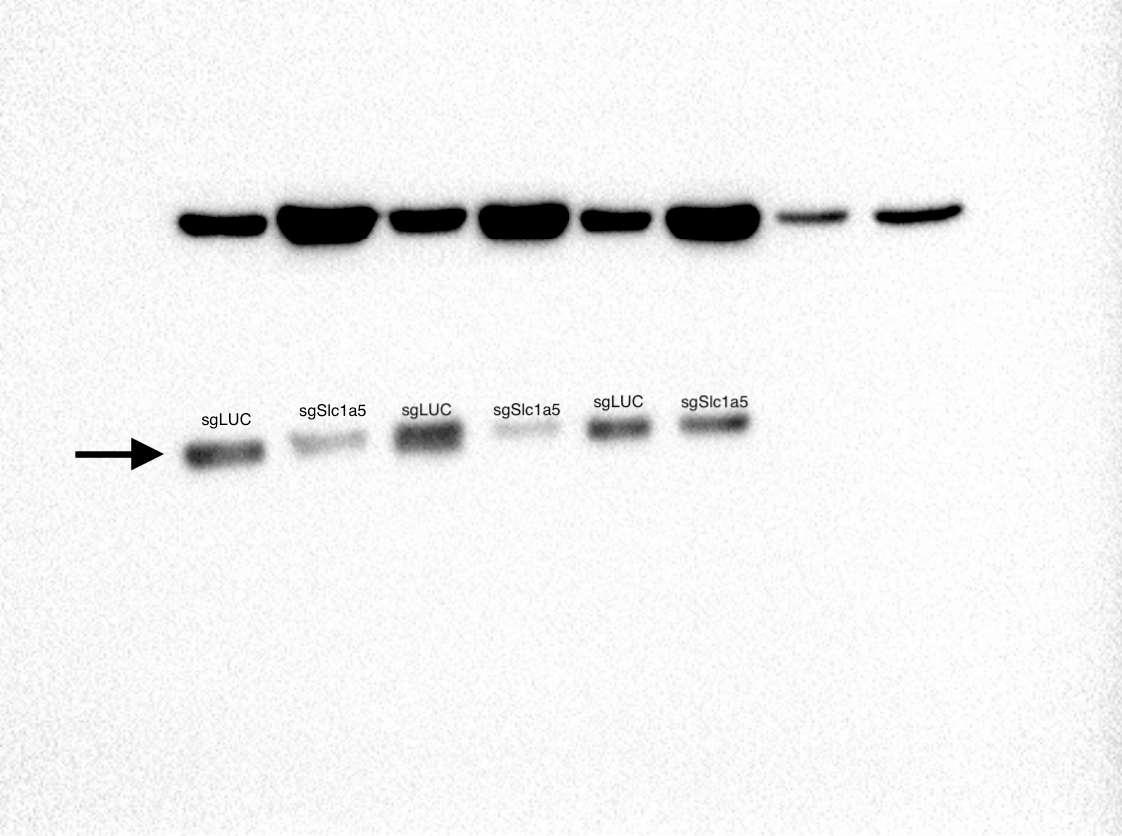

Supplement: Figure 4—source data 1. [file elife-71595-fig4-data1.zip › Figure 4 Source Data/Sharma Figure 4C Source Data/Sharma Figure 4C Source File Phospho S6 240_244.tiff]

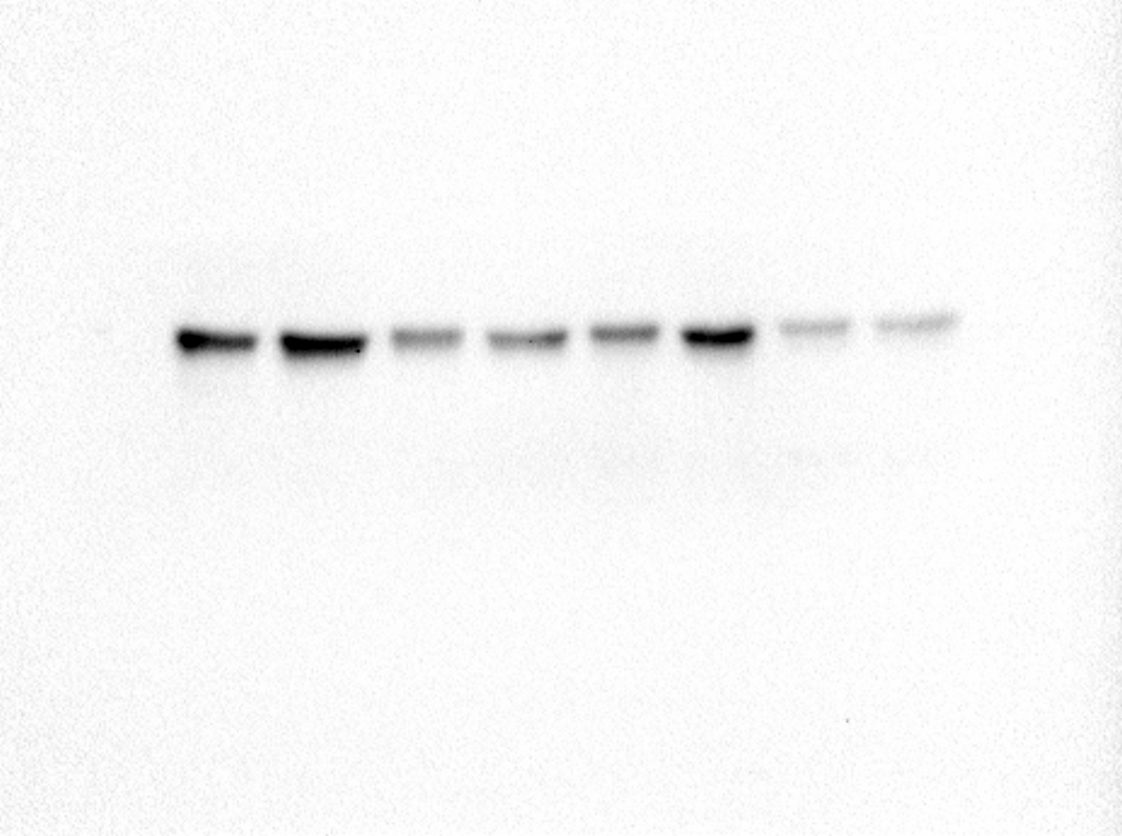

Supplement: Figure 4—source data 1. [file elife-71595-fig4-data1.zip › Figure 4 Source Data/Sharma Figure 4C Source Data/Sharma Figure 4C Source File phospho S51 EIF2A.tif]

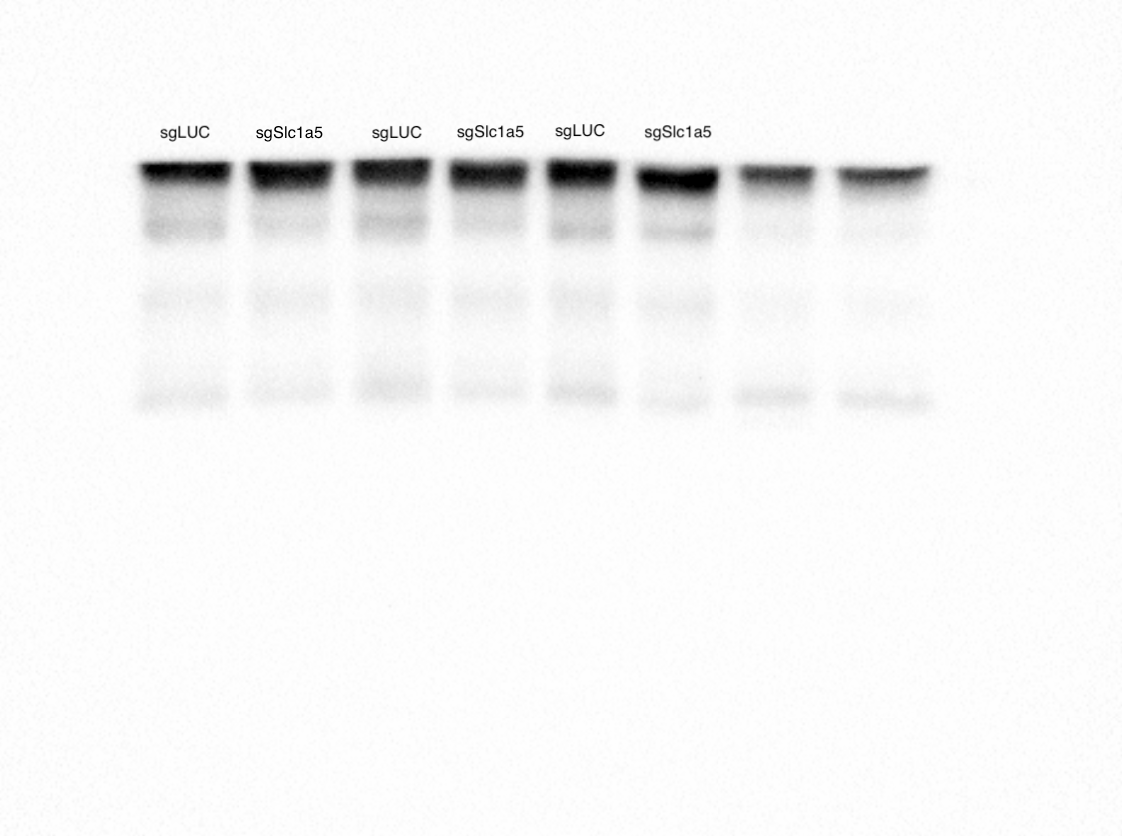

Supplement: Figure 4—source data 1. [file elife-71595-fig4-data1.zip › Figure 4 Source Data/Sharma Figure 4C Source Data/Sharma Figure 4C Source File Total Eif2a.tif]

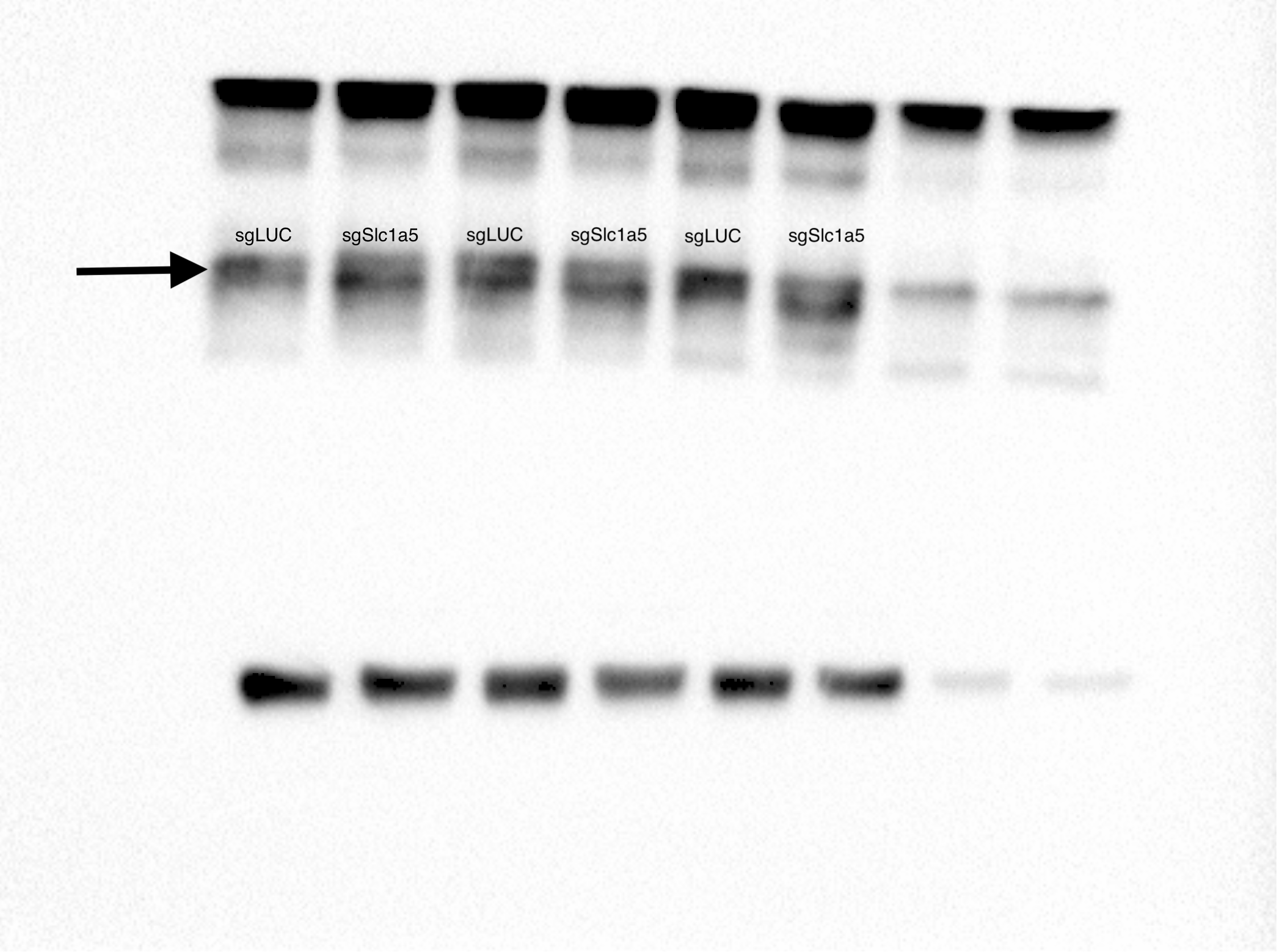

Supplement: Figure 4—source data 1. [file elife-71595-fig4-data1.zip › Figure 4 Source Data/Sharma Figure 4C Source Data/Sharma Figure 4C Source File total 4EBP1.tiff]

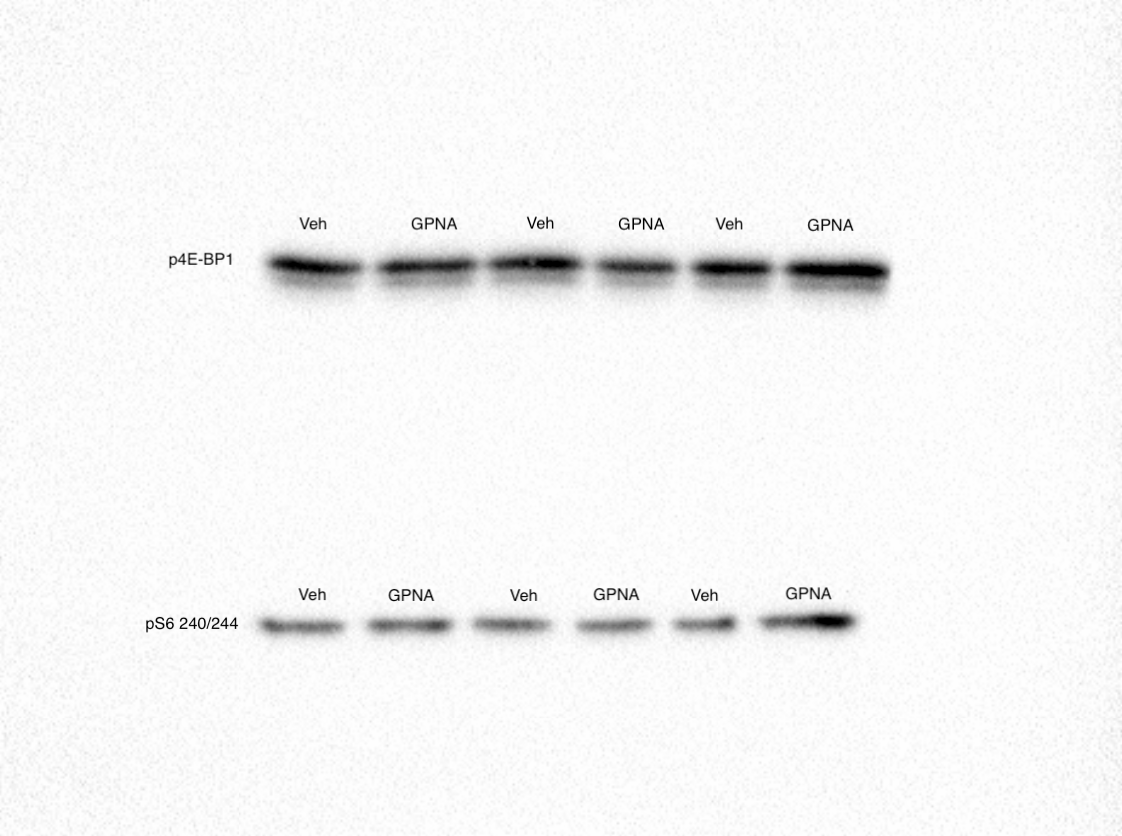

Supplement: Figure 4—figure supplement 1—source data 1. [file elife-71595-fig4-figsupp1-data1.zip › Figure 4 Figure supplement 1 Source Data/Sharma Figure 4 figure supplement 1C Source Data/Sharma Figure 2 figure supplement 2C Source File p4ebp1 top ps6 bottom.tiff]

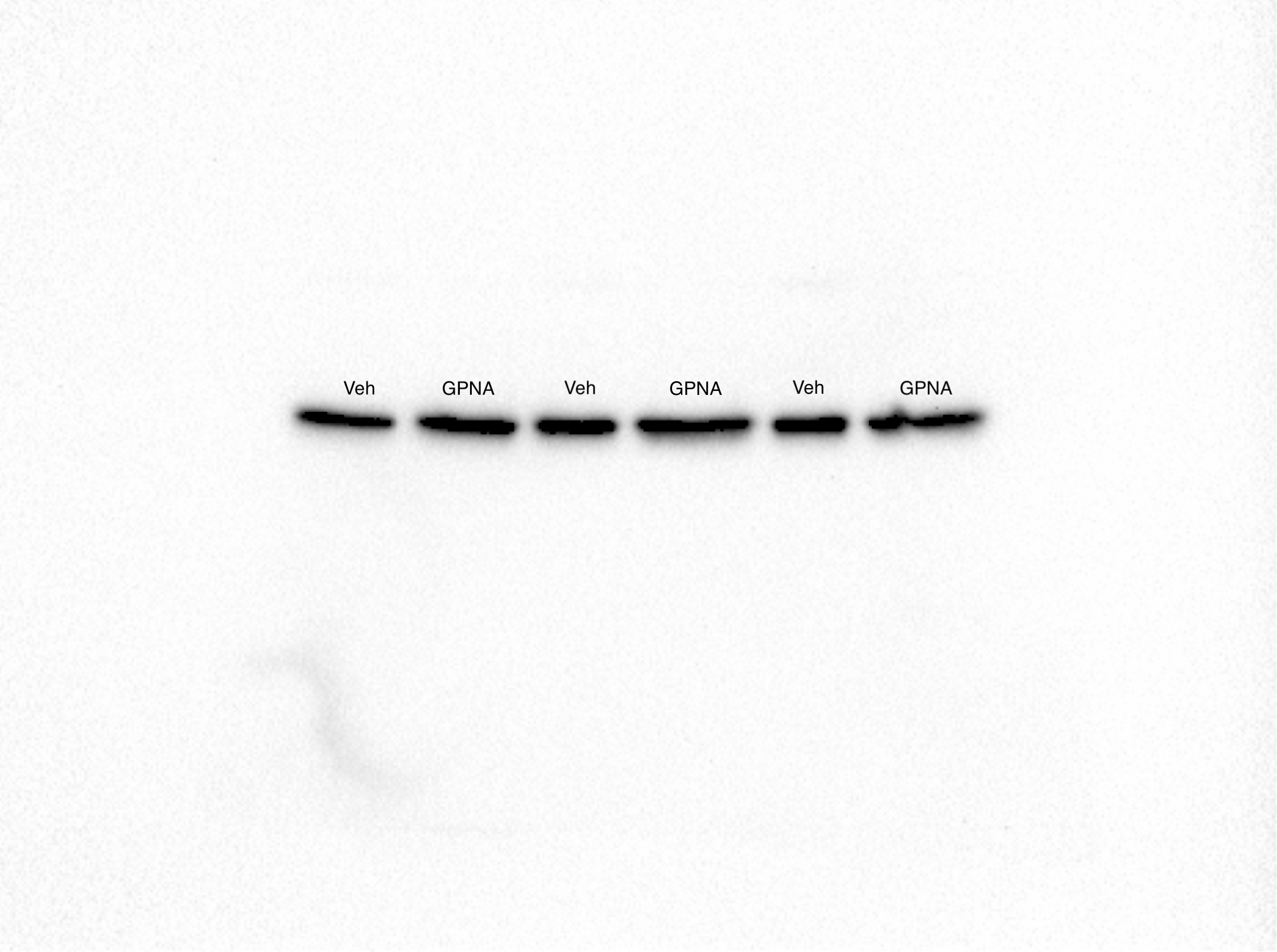

Supplement: Figure 4—figure supplement 1—source data 1. [file elife-71595-fig4-figsupp1-data1.zip › Figure 4 Figure supplement 1 Source Data/Sharma Figure 4 figure supplement 1C Source Data/Sharma Figure 2 figure supplement 2C Source File PEIF2A .tiff]

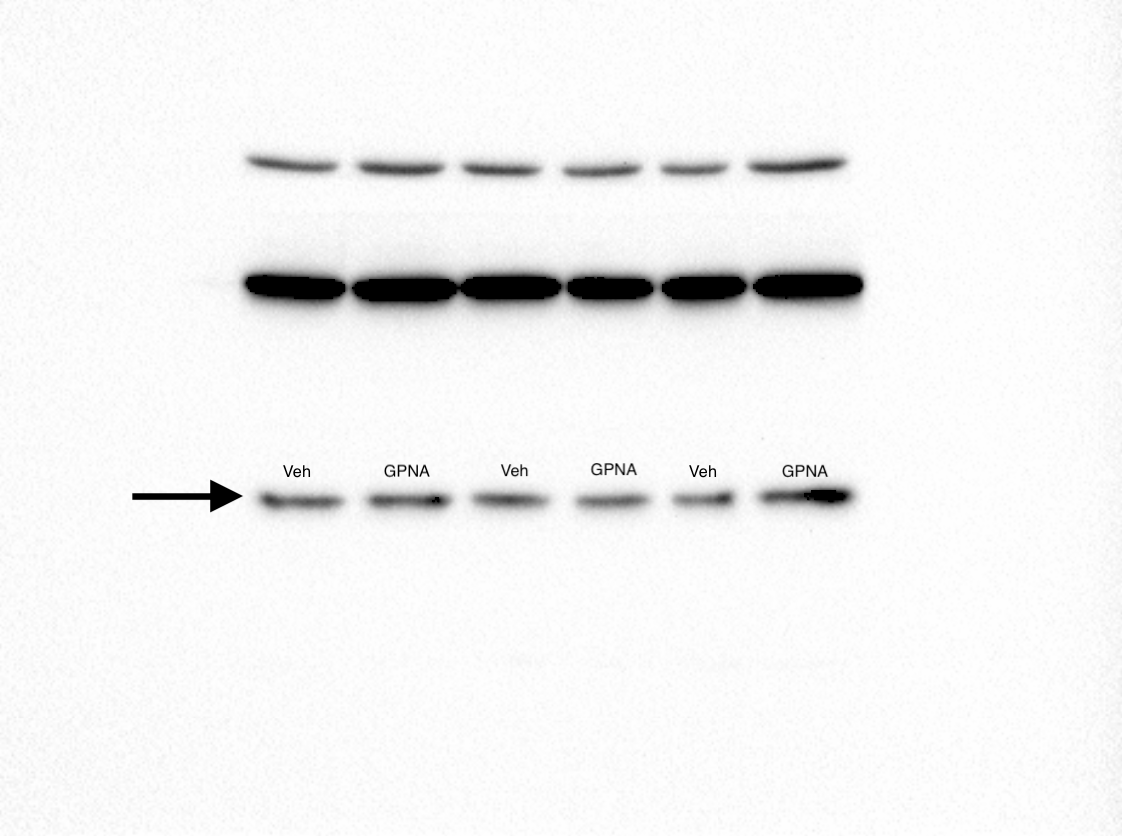

Supplement: Figure 4—figure supplement 1—source data 1. [file elife-71595-fig4-figsupp1-data1.zip › Figure 4 Figure supplement 1 Source Data/Sharma Figure 4 figure supplement 1C Source Data/Sharma Figure 2 figure supplement 2C Source File total S6.tiff]

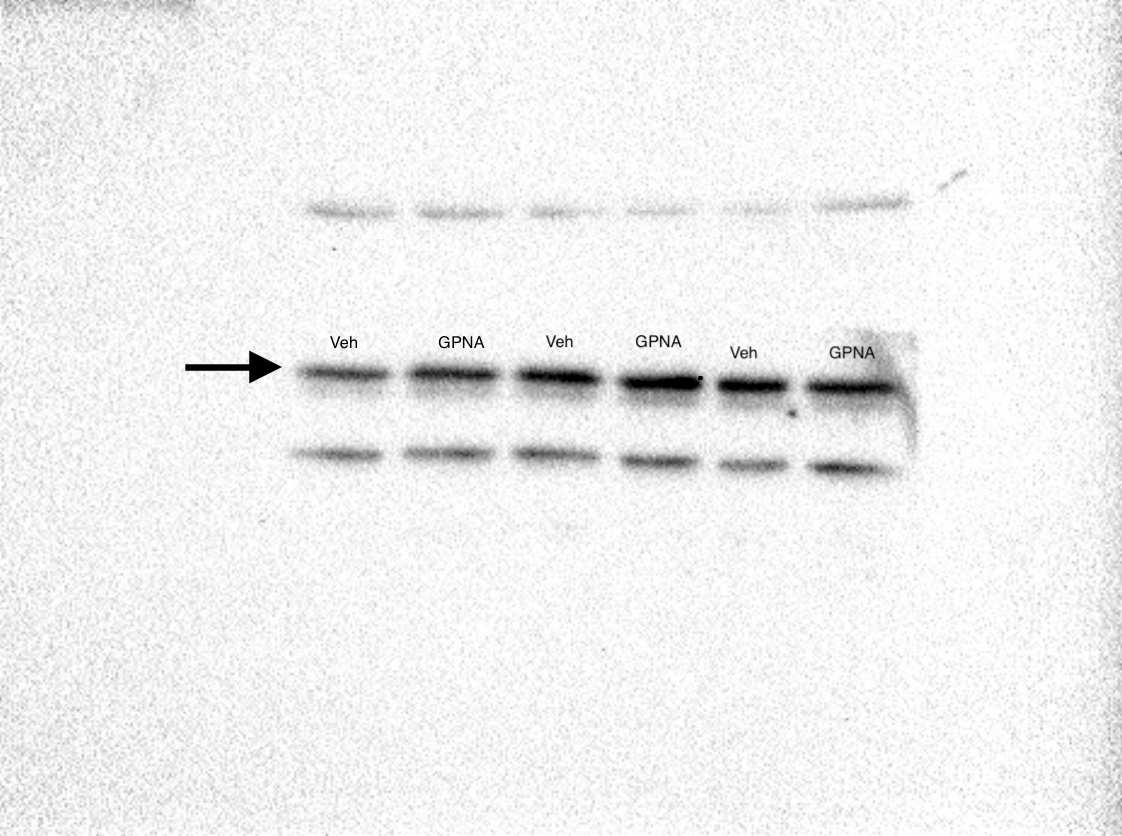

Supplement: Figure 4—figure supplement 1—source data 1. [file elife-71595-fig4-figsupp1-data1.zip › Figure 4 Figure supplement 1 Source Data/Sharma Figure 4 figure supplement 1C Source Data/Sharma Figure 2 figure supplement 2C Source Filetotal 4ebp1.tiff]

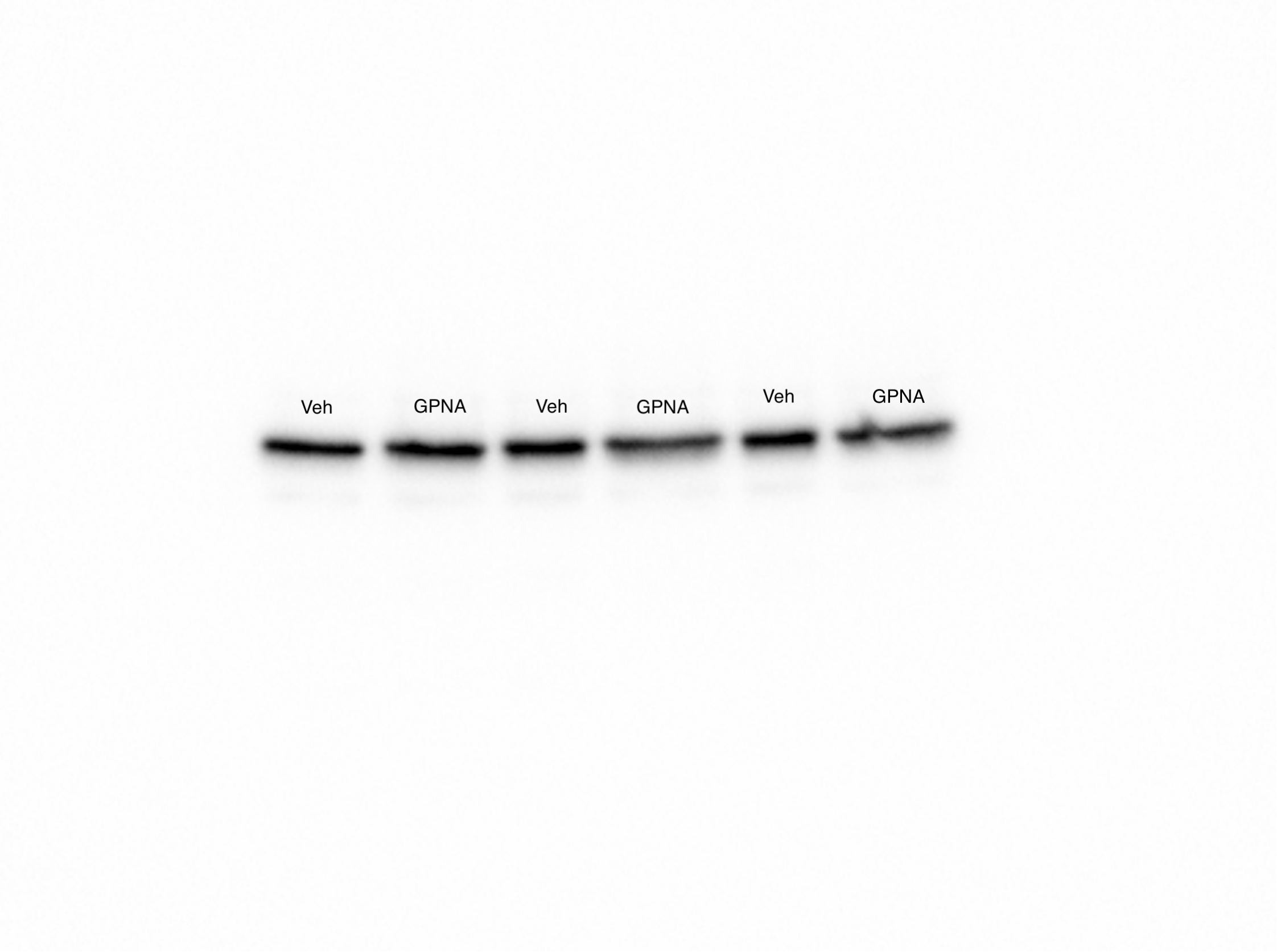

Supplement: Figure 4—figure supplement 1—source data 1. [file elife-71595-fig4-figsupp1-data1.zip › Figure 4 Figure supplement 1 Source Data/Sharma Figure 4 figure supplement 1C Source Data/Sharma Figure 2 figure supplement 2C Source File total eif2a.tiff]

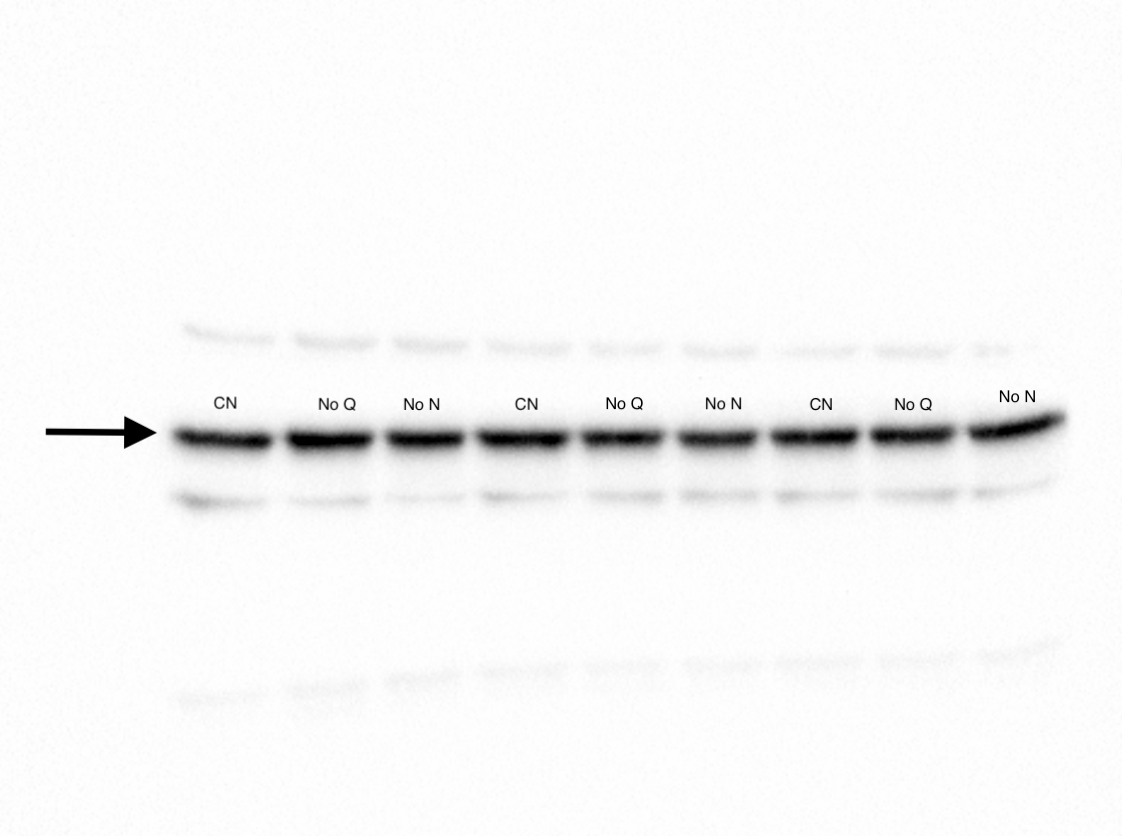

Supplement: Figure 5—source data 1. [file elife-71595-fig5-data1.zip › Figure 5 Source data/Sharma Figure 5C Source Data/Sharma Fig 5C Source Data total eif2a.tiff]

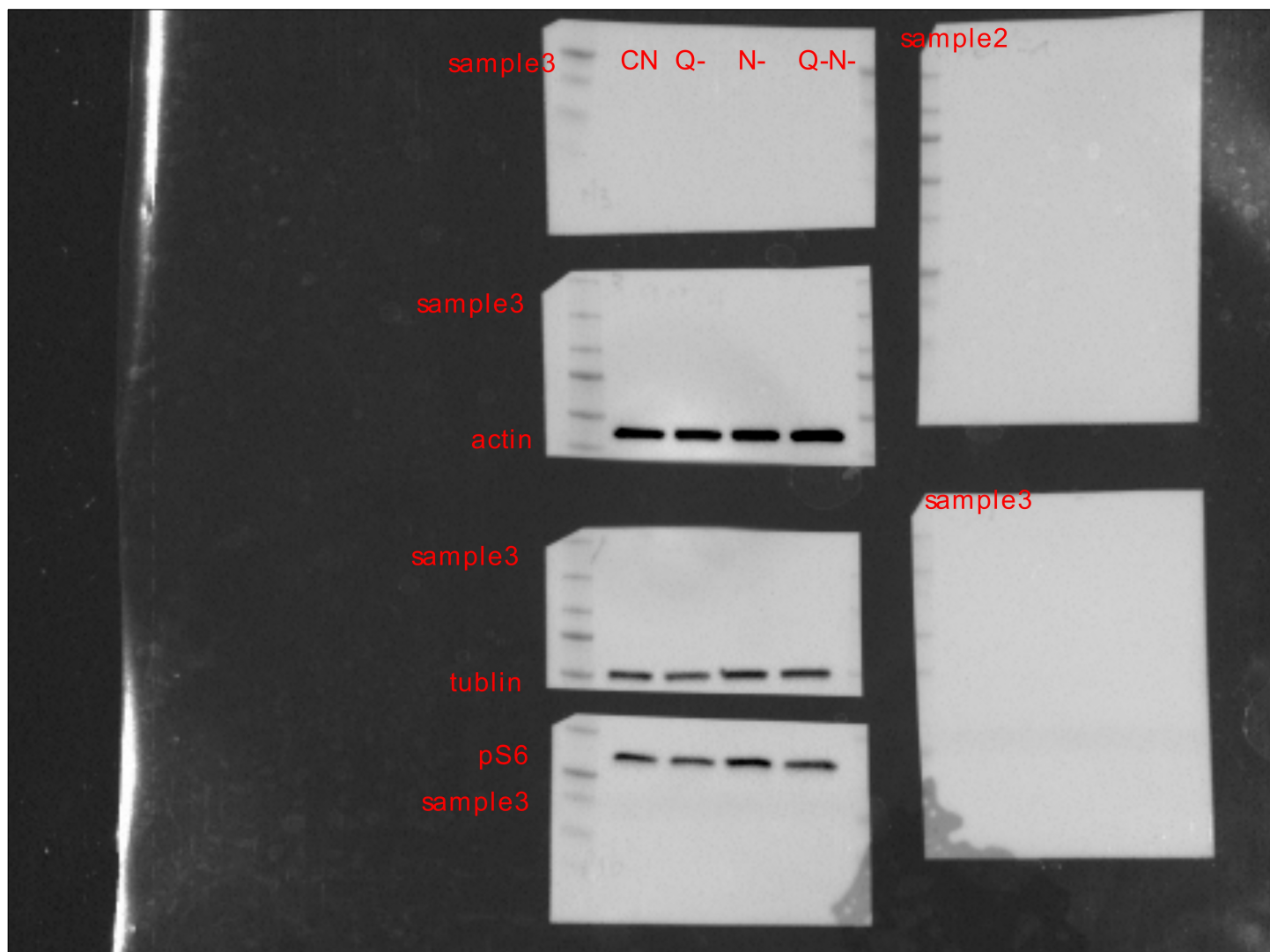

Supplement: Figure 5—source data 1. [file elife-71595-fig5-data1.zip › Figure 5 Source data/Sharma Figure 5C Source Data/Sharma Fig 5C Source Data phosphoS6 092220 sample 3.pdf]

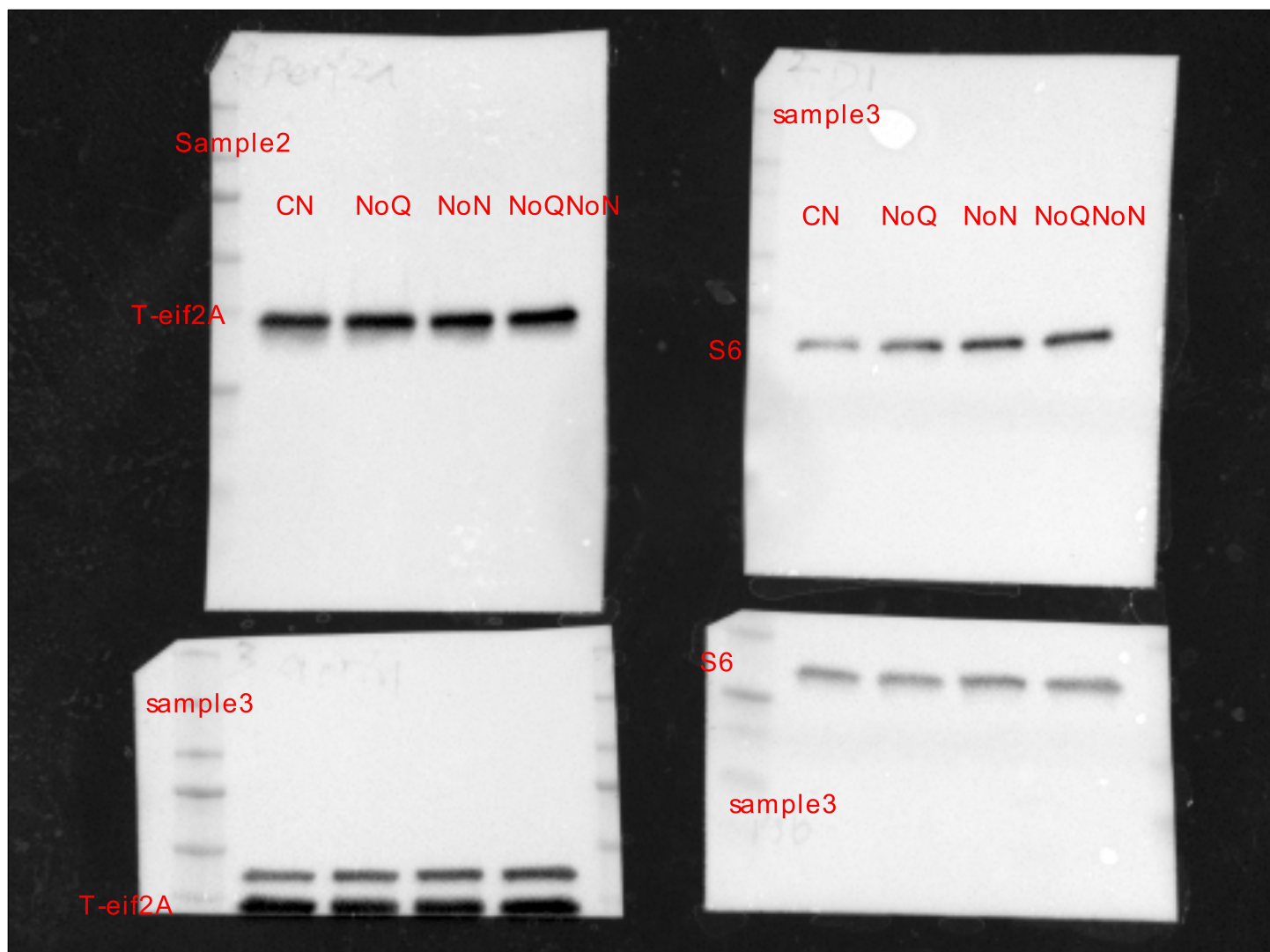

Supplement: Figure 5—source data 1. [file elife-71595-fig5-data1.zip › Figure 5 Source data/Sharma Figure 5C Source Data/Sharma Fig5C Source Data total S6 2sec 100120.pdf]

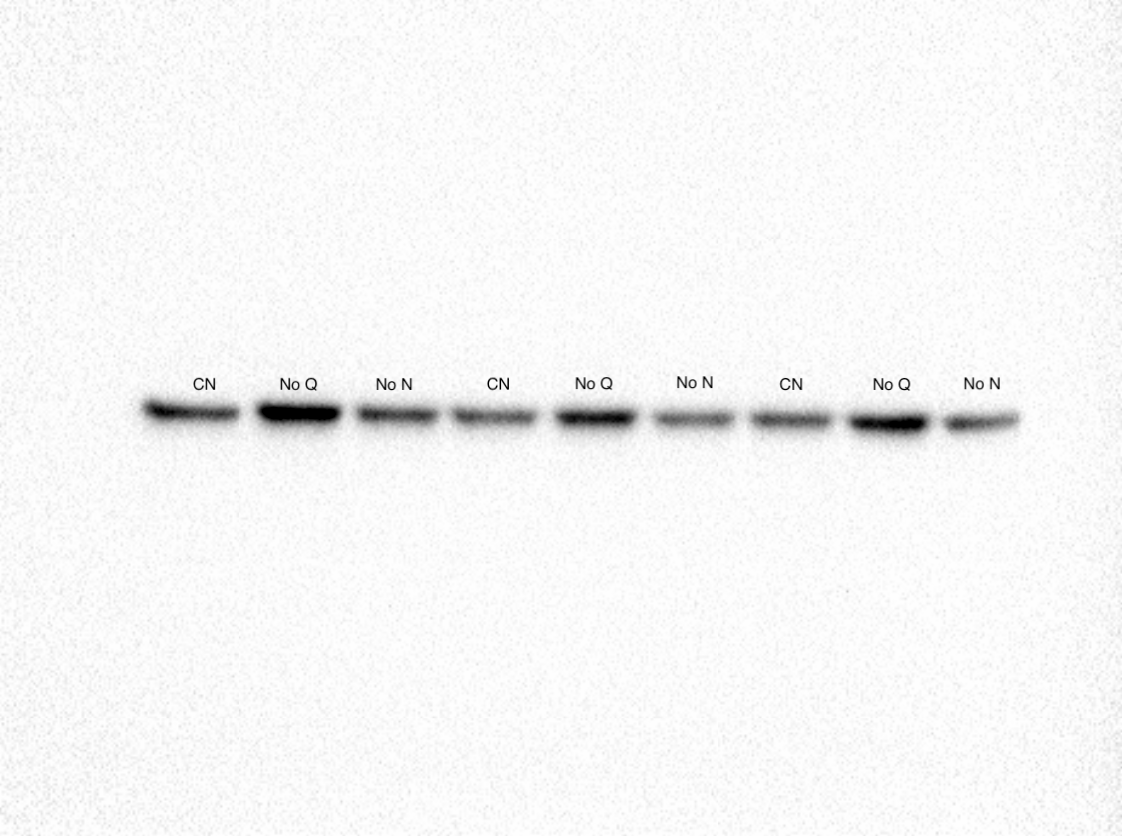

Supplement: Figure 5—source data 1. [file elife-71595-fig5-data1.zip › Figure 5 Source data/Sharma Figure 5C Source Data/Sharma Fig 5C Source Data peif2a .tiff]

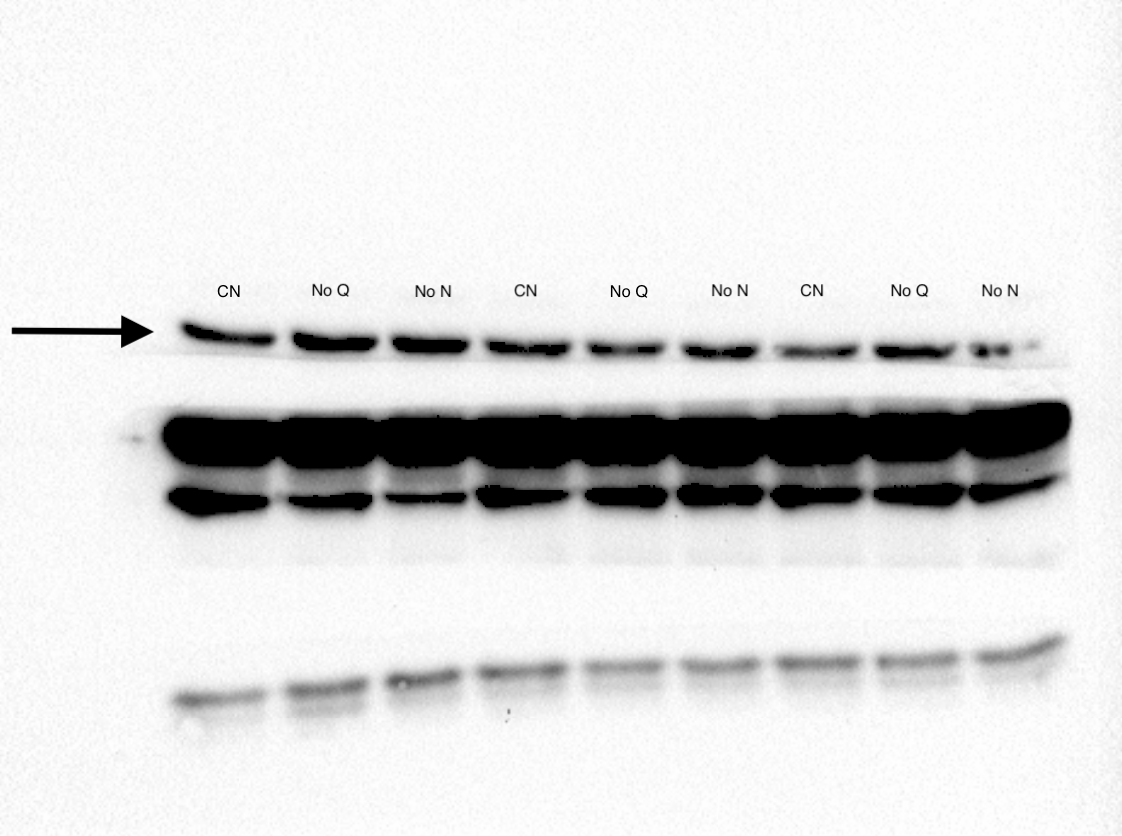

Supplement: Figure 5—source data 1. [file elife-71595-fig5-data1.zip › Figure 5 Source data/Sharma Figure 5C Source Data/Sharma Fig 5C Source Data ACTIN.tiff]

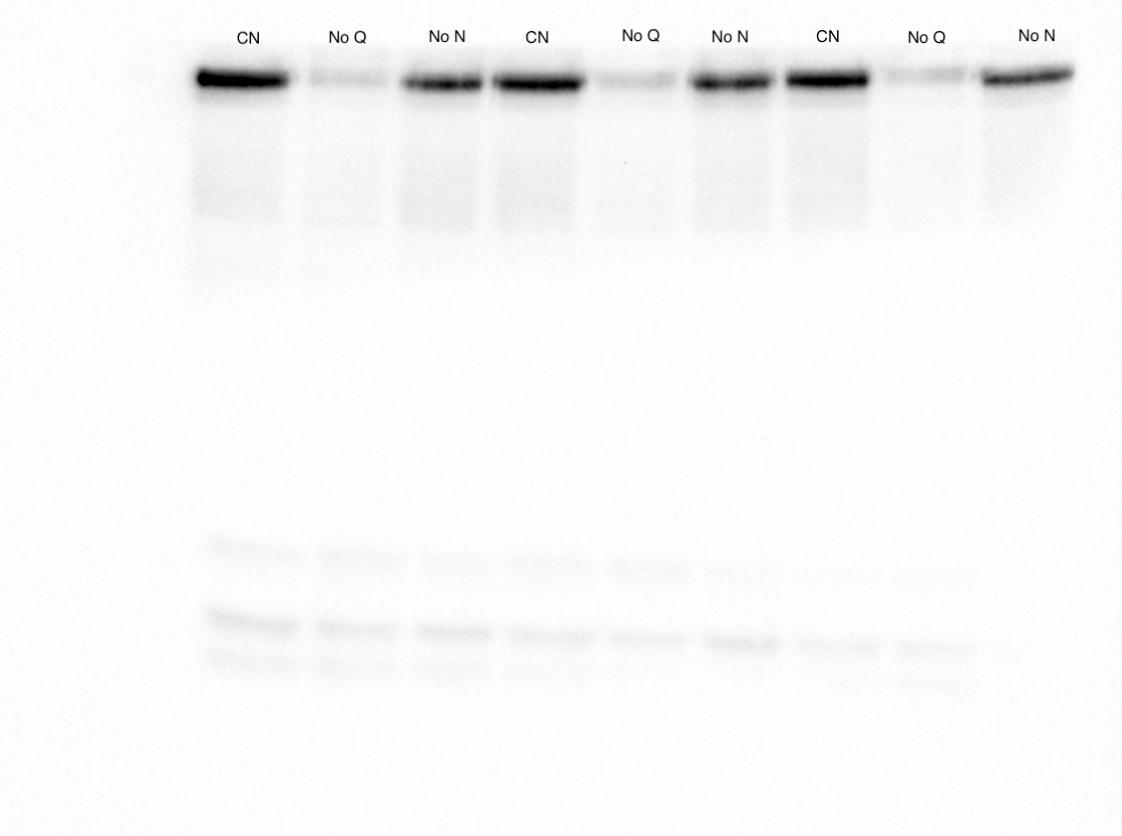

Supplement: Figure 5—source data 1. [file elife-71595-fig5-data1.zip › Figure 5 Source data/Sharma Figure 5C Source Data/Sharma Fig 5C Source Data Col1a1.tiff]

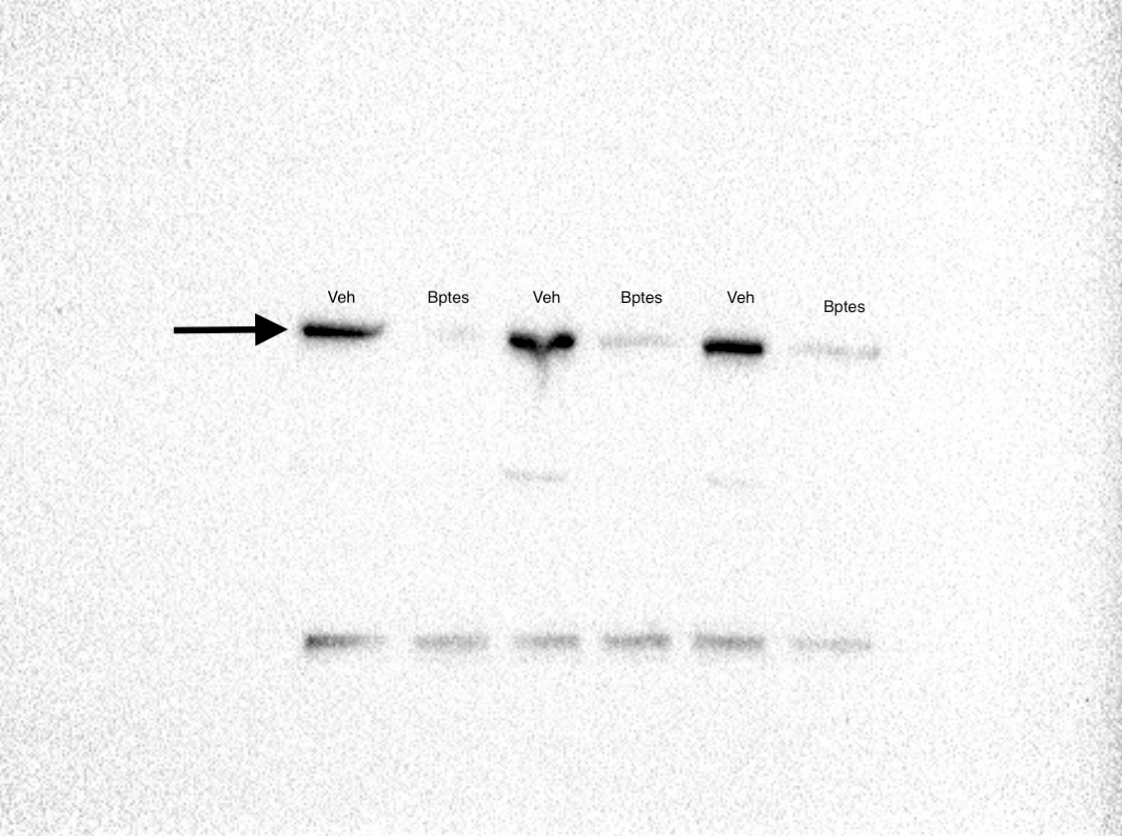

Supplement: Figure 6—source data 1. [file elife-71595-fig6-data1.zip › Figure 6 Source Data/Sharma Figure 6H Source Data/Sharma Fig 6H Source Data Col1a1 .tiff]

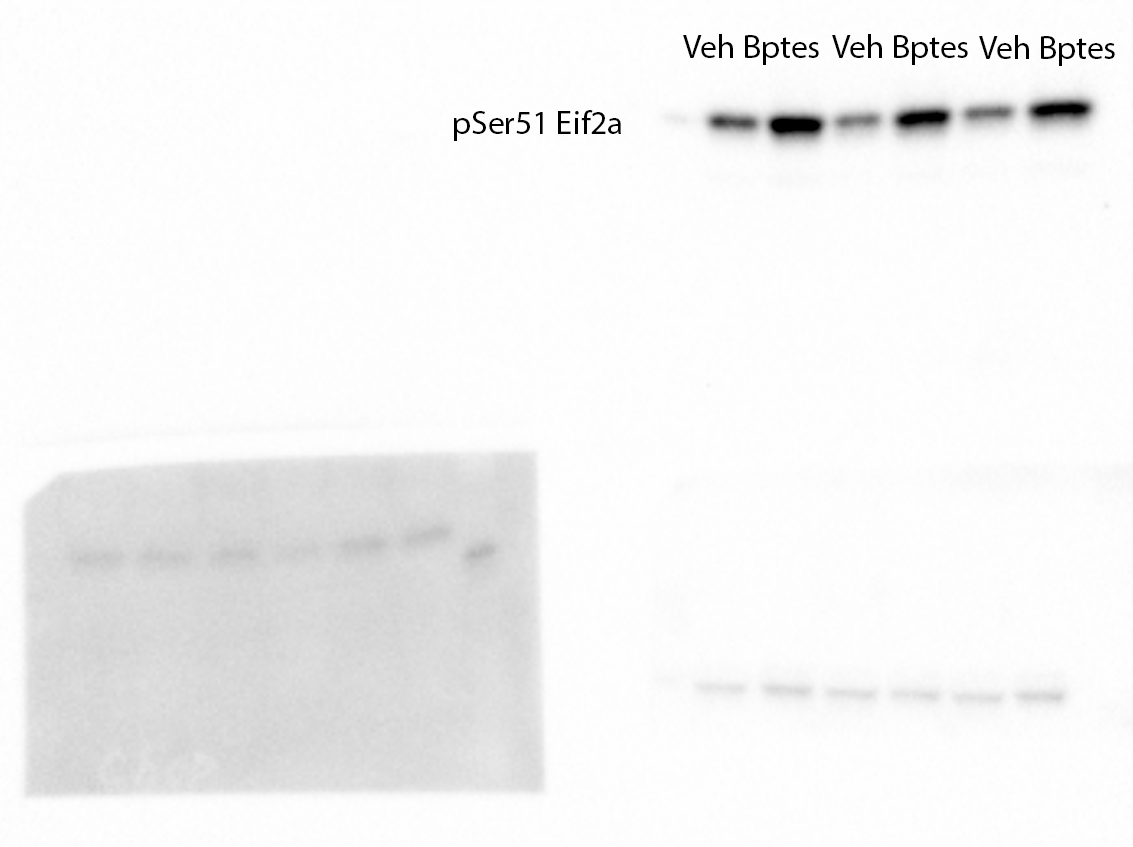

Supplement: Figure 6—source data 1. [file elife-71595-fig6-data1.zip › Figure 6 Source Data/Sharma Figure 6H Source Data/Sharma Fig 6H Source Data p-eif2a.tif]

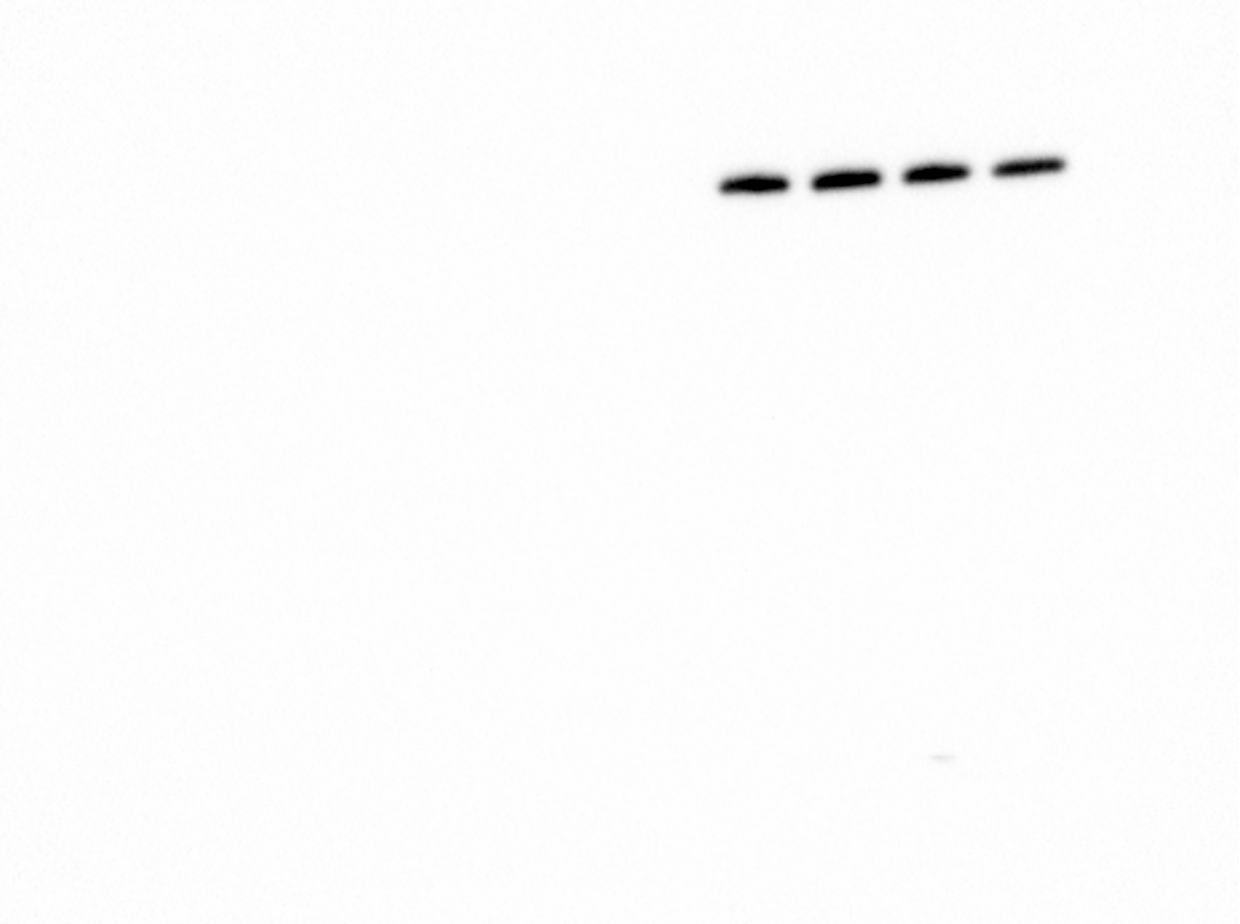

Supplement: Figure 6—source data 1. [file elife-71595-fig6-data1.zip › Figure 6 Source Data/Sharma Figure 6H Source Data/Sharma Fig 6H Source Data total S6.tif]

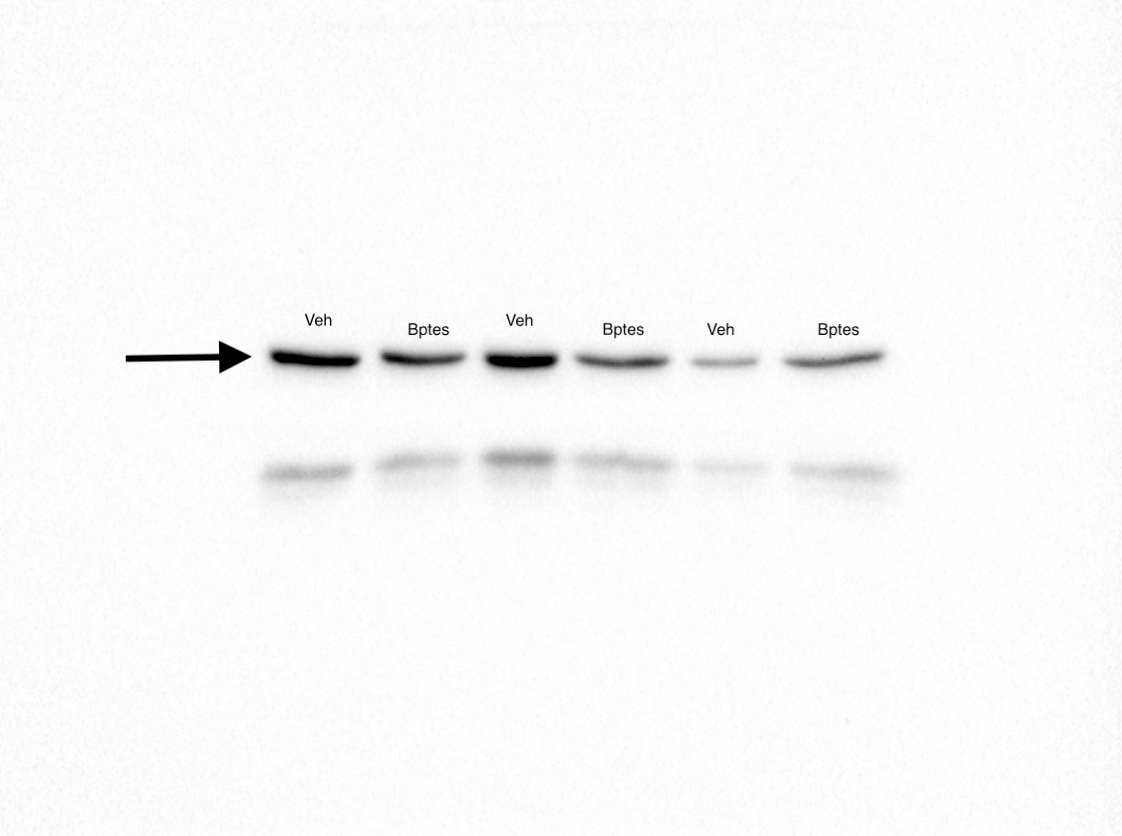

Supplement: Figure 6—source data 1. [file elife-71595-fig6-data1.zip › Figure 6 Source Data/Sharma Figure 6H Source Data/Sharma Fig 6H Source Data actin.tiff]

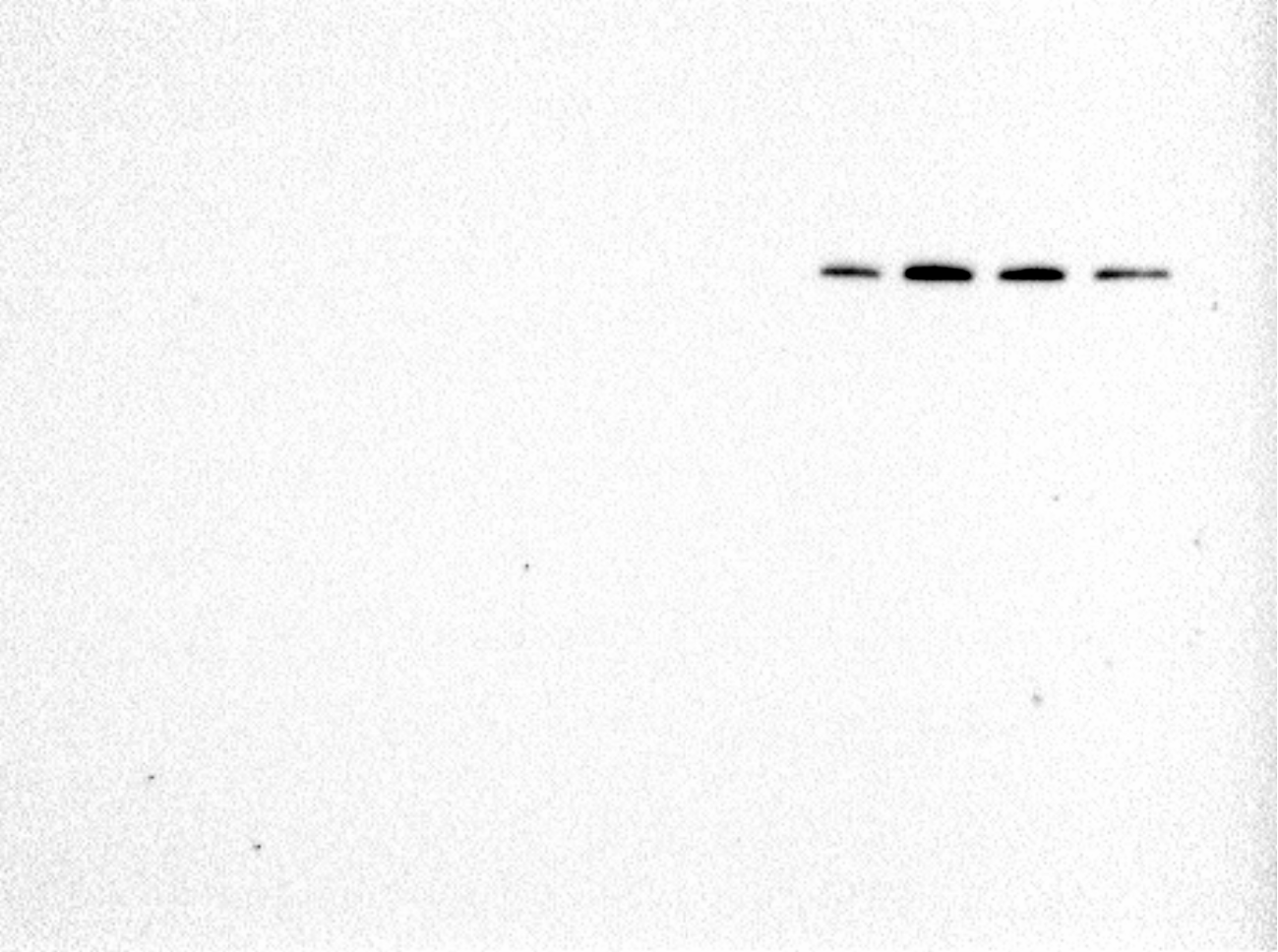

Supplement: Figure 6—source data 1. [file elife-71595-fig6-data1.zip › Figure 6 Source Data/Sharma Figure 6H Source Data/Sharma Fig 6H Source Data phospho Ser 240:244 S6.tif]

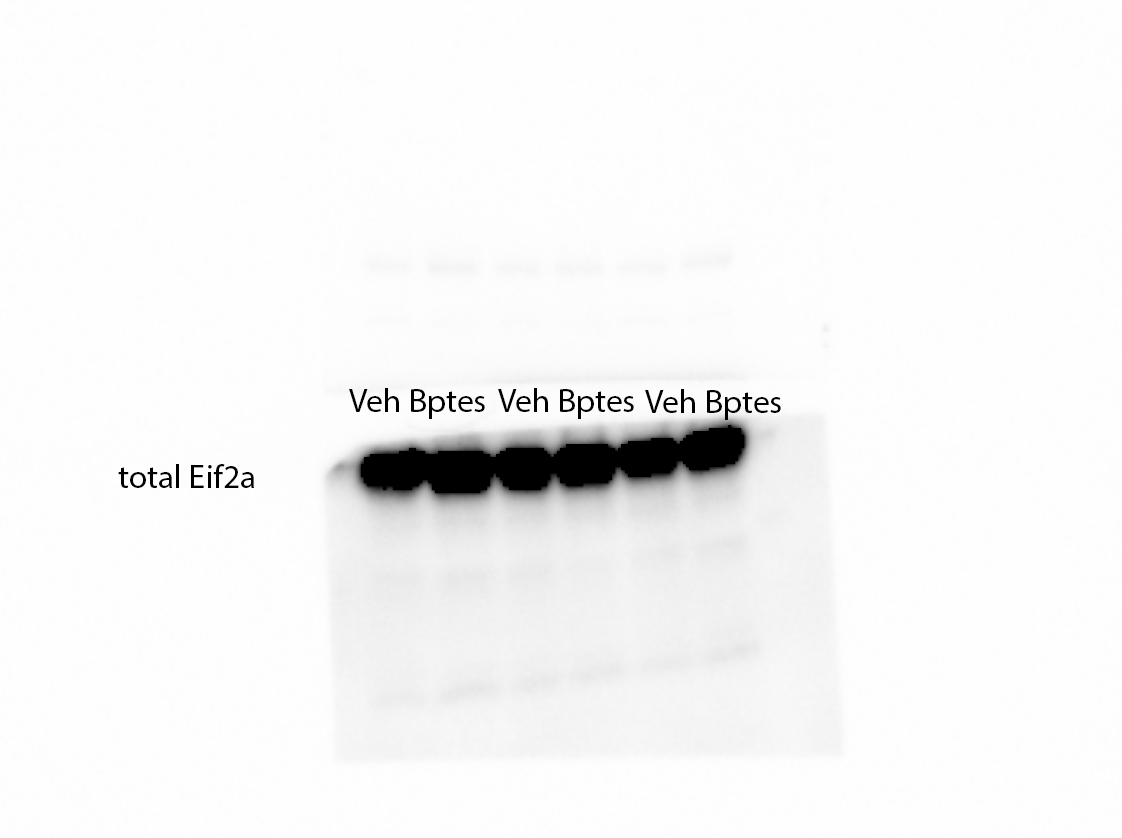

Supplement: Figure 6—source data 1. [file elife-71595-fig6-data1.zip › Figure 6 Source Data/Sharma Figure 6H Source Data/Sharma Fig 6H Source Data total Eif2a.tif]

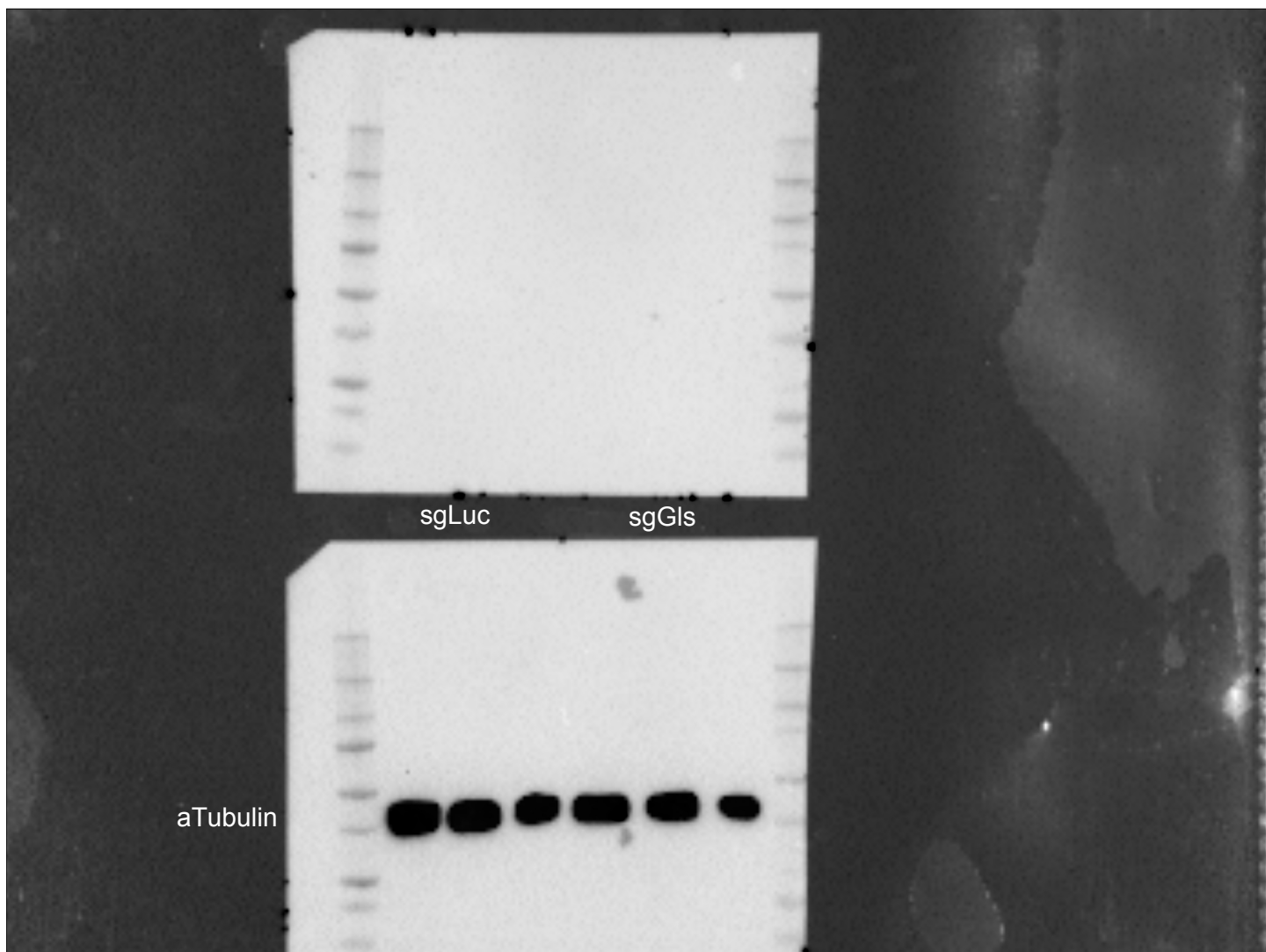

Supplement: Figure 6—figure supplement 1—source data 1. [file elife-71595-fig6-figsupp1-data1.zip › Figure 6 figure supplement 1 Source Data/Sharma Figure 6 figure supplement 1 Source Data/Sharma Figure 6 figure supplement 1 Source Data aTub.pdf]

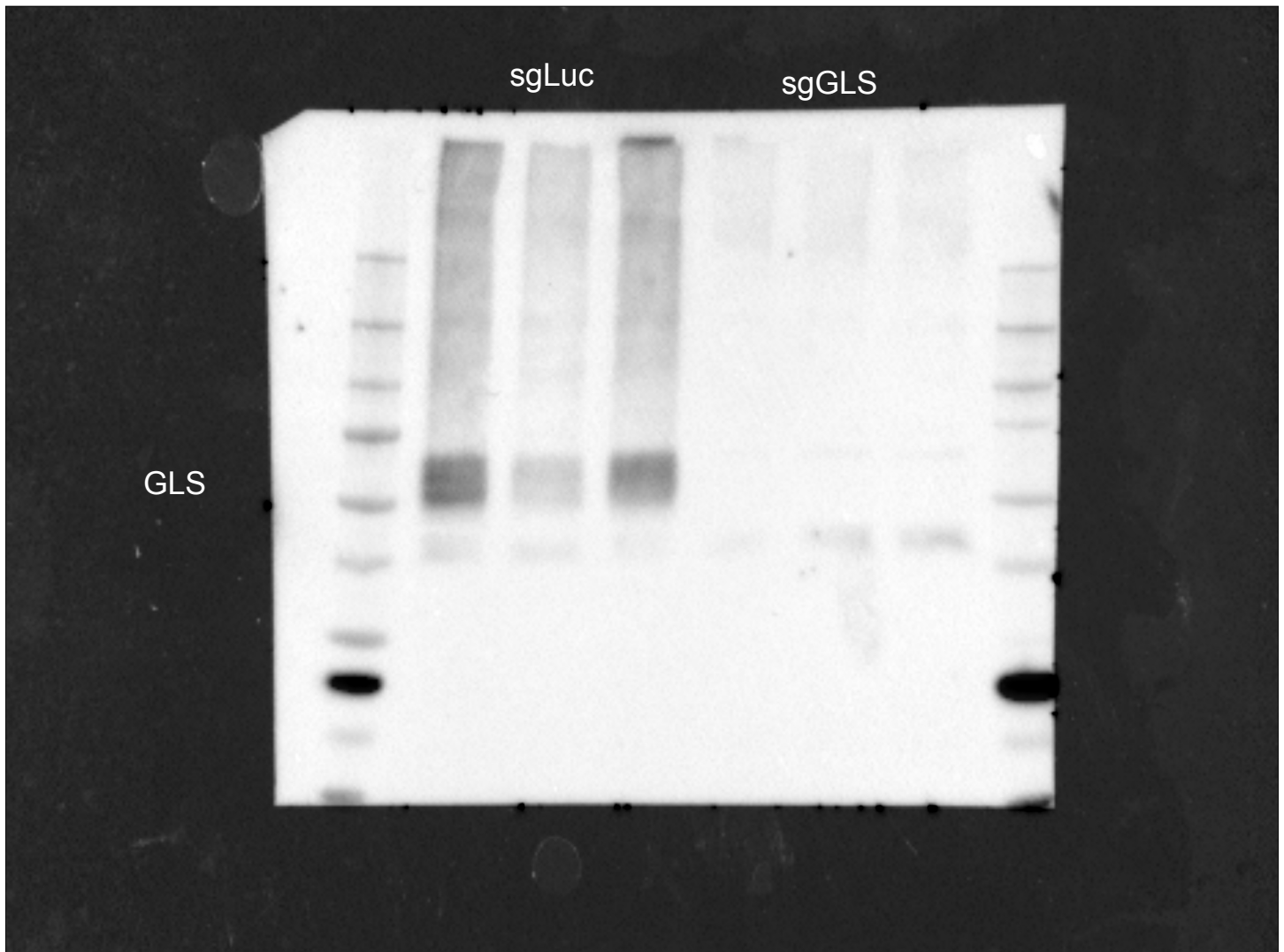

Supplement: Figure 6—figure supplement 1—source data 1. [file elife-71595-fig6-figsupp1-data1.zip › Figure 6 figure supplement 1 Source Data/Sharma Figure 6 figure supplement 1 Source Data/Sharma Figure 6 figure supplement 1 Source Data GLS Blot.pdf]
